# P-2042. Policy Matters – Standardizing Practice for Vancomycin Dosing in a Veteran Affairs Hospital

**DOI:** 10.1093/ofid/ofaf695.2206

**Published:** 2026-01-11

**Authors:** Parmida Parvaz, Sage Hendrickson, Allason Pantano, Milner Staub

**Affiliations:** Tennessee Valley Healthcare System, Nashville, Tennessee; Tennessee Valley Veterans Affairs Healthcare System, Nashville, Tennessee; VA Tennessee Valley Healthcare System, Nashville, Tennessee; VA Tennessee Valley Healthcare System; Vanderbilt University Medical Center, Nashville, Tennessee

## Abstract

**Background:**

Intravenous (IV) vancomycin management has dramatically changed since the 2020 American Journal of Health-System Pharmacist guidelines recommended area under the curve to minimum inhibitory concentration (AUC/MIC) as the optimal dosing strategy. This prompted widespread transitions in institutional dosing practices from trough-based to AUC/MIC-based strategies. The Veterans Affairs Tennessee Valley Healthcare System (VA TVHS) aimed to assess the extent of individual practice change and comfort with AUC/MIC vancomycin dosing at VA TVHS in the setting of increased evidence and available clinical tools but in the absence of an official institutional pharmacokinetic (PK) policy outlining IV vancomycin AUC/MIC dosing.

**Methods:**

To assess current practice and readiness for change from trough to AUC/MIC-based vancomycin dosing, a 10-item multiple-choice electronic REDCap survey was emailed to all clinical pharmacists who dose IV vancomycin at our facility. This included clinical medicine, critical care, bone marrow transplant, emergency department, and Community Living Center pharmacists who practice at one of the two VA facilities incorporated in VA TVHS. Descriptive statistics were performed in REDCap.

**Results:**

Among 34 pharmacists who received the survey link, 24 (71%) responded, of which 17 (70%) dose vancomycin daily. Dosing practice varied with 7 pharmacists (29%) reporting using trough-based, 3 (13%) using AUC/MIC, and 13 (54%) using a combination of both strategies. To determine maintenance dosing, 20 (83%) pharmacists use a PK online calculator as part of their approach, of which 18 (90%) used VancoPK.com©. While 13 (54%) were very or somewhat ready, 9 (38%) were completely or somewhat unready to completely transition to AUC/MIC dosing.

**Conclusion:**

Our data show that at VA TVHS, without a clear institutional policy, adoption of best practice and comfort in vancomycin dosing practice were highly variable. This can lead to inconsistencies in patient care and a potential for adverse events. Future work will assess the time to change in practice and comfort level after policy implementation.

**Disclosures:**

Milner Staub, MD, Gilead Sciences: Stocks/Bonds (Public Company)|Johnson & Johnson: Stocks/Bonds (Public Company)

